# Recurrent Tumor in Colorectal Cancer Requiring Combined Resection of Iliac or Femoral Vessels: Report of Four Cases

**DOI:** 10.70352/scrj.cr.24-0159

**Published:** 2025-05-01

**Authors:** Kentaro Abe, Hiroaki Nozawa, Katsuyuki Hoshina, Toshio Takayama, Kazuhito Sasaki, Koji Murono, Shigenobu Emoto, Yuichiro Yokoyama, Kensuke Kaneko, Takuro Shirasu, Shinya Abe, Yuzo Nagai, Masaru Kimura, Takahide Shinagawa, Yuichi Tachikawa, Satoshi Okada, Munetoshi Hinata, Akiko Takase, Tetsuo Ushiku, Soichiro Ishihara

**Affiliations:** 1 Department of Surgical Oncology, Graduate School of Medicine, The University of Tokyo, Tokyo, Japan; 2 Department of Vascular Surgery, Graduate School of Medicine, The University of Tokyo, Tokyo, Japan; 3 Department of Pathology, Graduate School of Medicine, The University of Tokyo, Tokyo, Japan

**Keywords:** recurrent tumor, colorectal cancer, combined resections, vascular invasion

## Abstract

**INTRODUCTION:**

Recurrent tumors in colorectal cancer may be removed along with adjacent blood vessels to achieve R0 resection. However, it remains unclear whether to aggressively perform this procedure because it may cause serious intraoperative or postoperative complications.

**CASE PRESENTATION:**

In Case 1, a 62-year-old man underwent radical surgery for rectosigmoid cancer. Three years later, computed tomography scans revealed a disseminated nodule near the left external iliac vessels. We resected the tumor and vessels that were reconstructed by bypass surgery. Histologically, the margins of the tumor were in contact with the adventitia of the vessels. In Case 2, a 63-year-old man underwent radical surgery for ascending colon cancer. A nodule was detected at the right iliac fossa 16 years later and appeared to invade the right femoral vessels. After systemic chemotherapy, the nodule was removed with partial resection of the right femoral artery and vein that were reconstructed by end-to-end anastomosis and bypass surgery, respectively. Histologically, the tumor was located 0.7 mm from the vessels. In Case 3, a 67-year-old woman underwent radical multivisceral resection for obstructive rectosigmoid cancer invading the adjacent organs. Fifteen months later, she developed local recurrence and subsequently received chemotherapy. She underwent en bloc resection of the tumor and the left internal iliac artery (IIA) near the bifurcation. The left external iliac artery was reconstructed by end-to-end anastomosis. Direct invasion of the IIA was proven histologically. In Case 4, a 74-year-old woman underwent radical surgery for ascending colon cancer with high microsatellite instability. Eight months later, a recurrent tumor was detected near the right external iliac vessels. After pembrolizumab and chemoradiotherapy, we resected the tumor and part of the external iliac vein; the defect was primarily closed with sutures. No viable tumor cells were found in the specimen. During the follow-up period (median: 52 months), 3 patients were alive without vascular surgery-related complications.

**CONCLUSIONS:**

It is difficult to accurately evaluate whether a recurrent tumor from colorectal cancer directly invades vessels using preoperative imaging. However, the combined resection of recurrent tumor and vessels may be required to achieve R0 resection, considering a short distance even in invasion-negative cases.

## Abbreviations

5-FU
: 5-fluorouracil
CAPOX
: capecitabine-oxaliplatin
CIA
: common iliac artery
CRT
: chemoradiation therapy
CT
: computed tomography
DVT
: deep vein thrombosis
EIA
: external iliac artery
EIV
: external iliac vein
FOLFIRI
: fluorouracil + levofolinate + irinotecan
FOLFOX
: fluorouracil + levofolinate + oxaliplatin
IIA
: internal iliac artery

## INTRODUCTION

In 2024, 2001140 new cancer cases and 611720 cancer deaths are projected to occur in the United States. Of these, colorectal cancer is currently the leading cause of death in men and the second leading cause in women.^1)^ It is also the second leading cause of death by malignancy in women and the second leading cause in men in Japan.^2)^ Locoregional recurrence has been documented in 4%–11.5% of patients after curative resection for colon cancer.^3–5)^ Recurrent tumor resection is considered for colorectal cancer in Japan when R0 resection is achievable. The long-term survival rate after the radical resection of recurrent colorectal cancer was previously reported to range between 15% and 40% at 20 years, and the achievement of R0 resection was identified as an extremely important prognostic factor.^6)^

Locoregional recurrence is classified as peri-anastomotic, mesenteric/paracolic (local lymph node disease), retroperitoneal (locoregional lymph node disease), and peritoneal, with the latter being the most common site.^5,7,8)^ Multivisceral resection may provide long-term survival in patients with locoregional recurrence of colorectal cancer.^9,10)^ In the case of adjacent vascular invasion, recurrent tumor resection with the associated resection of blood vessels may cause serious intraoperative or postoperative complications and require vascular reconstruction. A previous study reported no early deaths or severe complications after combined vessel resection and arterial reconstruction.^11)^ Nevertheless, there is insufficient consensus on the usefulness of the combined resection of blood vessels.

We herein investigated the clinicopathological characteristics of patients with recurrent colorectal cancer that required the combined resection of blood vessels in our hospital.

## CASE PRESENTATION

We retrospectively reviewed 4 consecutive patients who underwent recurrent tumor resection with the associated resection of iliac or femoral vessels for the intraperitoneal recurrence of colorectal cancer at the Department of Colorectal Surgery and Department of Vascular Surgery, the University of Tokyo, between 2008 and 2022. This was a retrospective analysis of data obtained for clinical purposes. Therefore, informed consent was replaced by the obligation of information to the participants and their right to opt out. Informed consent for the writing and publication of this case report was obtained from the patient. The present study was approved by the Ethics Committee of the University of Tokyo (No. 3252-[17]).

### Case 1

**Fig. 1:**
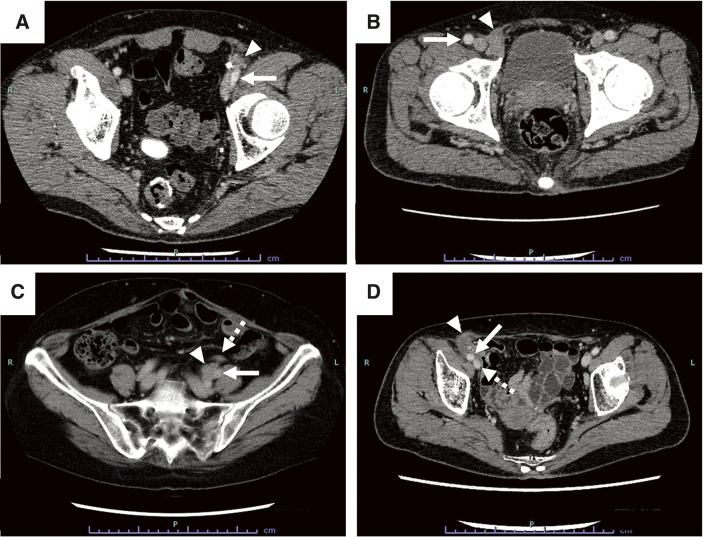
CT images showing a recurrent tumor with suspected vascular invasion in 4 cases. Arrowhead indicates the recurrent tumor in each case. (**A**) Case 1: An irregular nodule in contact with the left external iliac artery (arrow) was observed. (**B**) Case 2: An irregular nodule was observed in close proximity to the right femoral artery (arrow). (**C**) Case 3: A nodule was found near the left ureter (indicated by a dotted arrow), and in close proximity to the left internal iliac artery (arrow). (**D**) Case 4: A soft tissue nodule with calcification was located anterior to the right external iliac artery (arrow) and vein (dotted arrow). CT, computed tomography

**Fig. 2:**
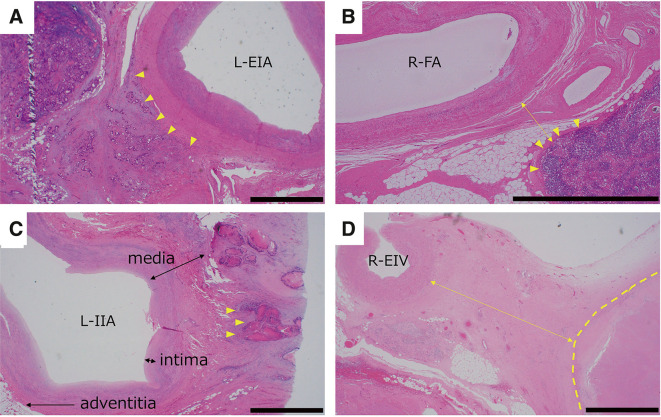
Hemoxylin and eosin staining of resected recurrent colorectal cancer with iliac or femoral vessels in 4 cases. Arrowheads indicate recurrent tumor margins. Bars indicate 2 mm. (**A**) Case 1: The recurrent tumor invaded into the border of the adventitia of the L-EIA. (**B**) Case 2: The recurrent tumor did not invade the R-FA; the distance between the recurrent tumor and R-FA was 0.7 mm (2-way arrow). (**C**) Case 3: The recurrent tumor invaded into the media of the L-IIA. Two-way arrows indicate the layers of the intima and media, and a 1-way arrow indicates the adventitia. (**D**) Case 4: Histologically, no viable tumor was found in the resected section. An area with necrosis and fibrosis (indicated by a dashed line) was in close proximity to the R-EIV. The shortest distance between the area and R-EIV was 4.3 mm (2-way arrow). L-EIA, left external iliac artery; L-EIV, left external iliac vein; L-IIA, left internal iliac artery; R-FA, right femoral artery

A 62-year-old man underwent laparoscopy-assisted high anterior resection for rectosigmoid cancer (pT4aN2M0 and pStage IIIB) and received 8 cycles of adjuvant capecitabine-oxaliplatin (CAPOX) chemotherapy. Two years later, he was diagnosed with recurrent peritoneal dissemination in the left lower abdomen and underwent laparoscope-assisted disseminated nodule resection. Although he received adjuvant CAPOX therapy again postoperatively, computed tomography (CT) revealed a disseminated tumor in close proximity to the left external iliac artery (EIA) and vein (EIV) at the end of chemotherapy. These vessels appeared to be invaded by the tumor (**Fig. 1A**). We scheduled resection of the disseminated nodule, and the combined resection of external iliac vessels was planned if necessary. We performed en bloc resection of the tumor and the artery and vein that were reconstructed by bypass surgery using expanded-polytetrafluoroethylene grafts. Pathologically, the recurrent tumor made contact with the adventitia of the artery (**Fig. 2A**). Postoperative perfusion in the left lower limb was not complicated in contrast-enhanced CT scans. However, the patient developed lung metastasis and recurrence near the left femoral artery and died 4 years after the last surgery.

### Case 2

A 63-year-old man underwent right hemicolectomy for ascending colon cancer (pT4aN0M0 and pStage II) and received tegafur/uracil and calcium folinate as adjuvant chemotherapy. At the age of 68 years, he underwent hepatectomy for liver metastasis, followed by the same oral 5-fluorouracil (5-FU)-based regimen. Seven years later, he was diagnosed with left inguinal metastasis, which was surgically removed. He received 10 cycles of adjuvant CAPOX therapy followed by 5 cycles of capecitabine monotherapy. Four years later, CT showed a disseminated nodule that was suspected to involve the right femoral artery and vein (**Fig. 1B**). He received 11 cycles of FOLFOX (fluorouracil + levofolinate + oxaliplatin) plus bevacizumab as neoadjuvant chemotherapy. Due to hypersensitivity reactions to oxaliplatin, he then received 8 cycles of FOLFIRI (fluorouracil + levofolinate + irinotecan) plus bevacizumab. Since there were no new lesions during the above treatments, the nodule was resected in conjunction with partial abdominal wall resection, partial pubic bone resection, and resection of the right femoral vessels. The artery was reconstructed by end-to-end anastomosis, and the vein was reconstructed by bypass surgery using a gelatin-coated Dacron graft. Pathologically, the margin of the disseminated nodule was located 0.7 mm from the vessels (**Fig. 2B**). The patient has survived for 69 months without recurrence. Postoperative CT scans showed patency of the reconstructed vessels.

### Case 3

A 67-year-old woman underwent high anterior resection, partial resection of the terminal ileum, appendectomy, and right salpingo-oophorectomy for obstructive rectosigmoid cancer (pT4b [right fallopian tube] N1M0 and pStage IIIC). She received adjuvant oral 5-FU-based chemotherapy for 5 months. Five months later, she was diagnosed with local recurrence and received mFOLFOX6 and cetuximab for 3 months. Since she refused surgical resection at the time of tumor shrinkage, she continued to receive oral 5-FU-based chemotherapy for another 6 months. However, follow-up CT scans showed enlargement of the local recurrent tumor and its close proximity to the left common iliac artery (CIA) and internal iliac artery (IIA) (**Fig. 1C**). The tumor was also located in the proximity of the left ureter, causing hydronephrosis shortly thereafter. Preoperative ureteroscopy showed a mass in the left ureter, and a biopsied specimen revealed adenocarcinoma. We planned resection of the tumor together with the adjacent arteries and left ureter. The recurrent tumor appeared to involve the left ureter, left fallopian tube, and the root of the left IIA intraoperatively and, thus, we performed recurrent tumor resection, sigmoid colectomy, left nephroureterectomy, left salpingo-oophorectomy, and partial resection of the left IIA. As its proximal stump tore toward the left EIA during the operation, we attempted to make primary closure with a 4-0 polypropylene suture. However, we found weak pulsation and thrill in the left EIA. We decided to cut the narrow segment of the EIA and perform end-to-end anastomosis between the left CIA and EIA. Pathologically, the recurrent tumor invaded the media of the left IIA (**Fig. 2C** and **Supplementary Fig. 1**). Although the patient developed recurrence in the bladder and metastasis in the para-aortic lymph nodes, both recurrent lesions were resected. The patency of the reconstructed EIA was confirmed in follow-up CT images. She has survived for 55 months without recurrence after the final surgery.

### Case 4

A 74-year-old woman underwent laparoscopy-assisted right hemicolectomy for ascending colon cancer (pT4aN1aM0 and pStage IIIB) with high microsatellite instability. Eight months later, a 30-mm mass appeared in close proximity to the right external iliac vessels on CT scans. However, the mass remained unchanged in size for 3 years and was followed up because of her preference. Forty-six months after the primary surgery, the mass increased to 39 mm in size, being suspected to be a recurrent tumor. She received 6 cycles of neoadjuvant pembrolizumab, which reduced the size of the mass to 30 mm. However, the vascular invasion by the tumor still remained, and she next received chemoradiation therapy (CRT) using capecitabine and long-course irradiation (50.4 Gy/28 fr). Although CT scans showed further shrinkage of the tumor to 21 mm, it still appeared to invade the right EIA and EIV (**Fig. 1D**). We resected the recurrent tumor together with part of the right EIV that was primarily closed with 5-0 polypropylene sutures. In the specimen, no viable tumor was found with remaining necrotic and fibrotic tissues (**Fig. 2D**). Its border was 4.3 mm from the vessel. She has survived without recurrence for 21 months until the final visit.

We summarized the surgical outcomes of primary tumors in the present 4 cases in **Table 1**, and preoperative treatments and surgical procedures for recurrent tumors in **Table 2**. Three patients had received neoadjuvant treatments, except for Case 1, where the recurrent tumor was revealed at the end of adjuvant chemotherapy. All patients underwent vascular reconstruction.

**Table 1:** Surgical procedure, pathological diagnosis, and postoperative treatment for primary tumor

Case	Sex	Age (years)	Region	Procedure	LND	Margin status	Depth of invasion (p)	Lymphovascular invasion	pStage	Adjuvant chemotherapy
1	Male	62	RS	HAR	D3	R0	T4a	ly 2, v 1	IIIB	CAPOX
2	Male	63	A	RHC	D3	R0	T4a	ly 0, v 2	II	Tegafur/uracil + folinate
3	Female	67	RS	HAR	D3	R0	T4b (left fallopian tube)	ly 2, v 2	IIIC	Tegafur/uracil + folinate
4	Female	74	A	RHC	D3	R0	T4a	ly 2, v 2	IIIB	None

The histology of all cases is adenocarcinoma. pStage is based on the 9th tumor node metastasis (TNM) classification system for colorectal cancer.

A, ascending colon; CAPOX, capecitabine + oxaliplatin; HAR, high anterior resection; LND, lymph node dissection; RHC, right hemicolectomy; RS, rectosigmoid

**Table 2:** Preoperative treatment and surgical procedure for recurrent tumor

Case	Preoperative treatment	Procedure	Resected vessels	Repair of vessels
1	None	Disseminated nodule resection	Left EIA and EIV	Artery and vein: Bypass surgery using prosthetic graft
2	FOLFOX + Bev ↓ FOLFIRI + Bev	Disseminated nodule resection + partial abdominal wall resection + partial pubic resection	Right femoral artery and vein	Artery: End-to-end anastomosis
				Vein: Bypass surgery using prosthetic graft
3	mFOLFOX6 + Cet ↓ Tegafur/uracil + levofolinate	Resection of recurrent tumor + sigmoid colectomy + left nephroureterectomy + left salpingo-oophorectomy	Left IIA	End-to-end anastomosis between left CIA and EIA
4	CRT (capecitabine + pembrolizumab + 50.4 Gy/28 fr)	Resection of recurrent tumor + partial peritoneal resection	Right EIV	Primary closure

Bev, bevacizumab; Cet, cetuximab; CIA, common iliac artery; CRT, chemoradiation therapy; EIA, external iliac artery; EIV, external iliac vein; FOLFIRI, fluorouracil + levofolinate + irinotecan; mFOLFOX6, fluorouracil + levofolinate + oxaliplatin; IIA, internal iliac artery

The changes in the sizes of all recurrent tumors by preoperative treatments on CT scans were summarized in **Table 3**. The changes in tumor marker levels were also shown in **Table 4**. Although the changes in tumor marker levels were not consistent among patients, preoperative treatments reduced the tumor size in all cases.

**Table 3:** Change in recurrent tumor size (mm)

Case	Before neoadjuvant treatment	Before surgery (after neoadjuvant treatment) for recurrent tumor	Resected specimen
1	N.D.	22	30
2	31	30	75
3	45	19	23
4	39	21	25

N.D., no data

**Table 4:** Change in tumor markers

Case	Tumor markers	Before surgery for primary tumor	Before neoadjuvant treatment for recurrent tumor	Before surgery (after neoadjuvant treatment) for recurrent tumor	After surgery for recurrent tumor
1	CEA (ng/mL)	7.1	N.D.	10.4	4.5
	CA 19-9 (U/mL)	26.0	N.D.	29.0	28.0
2	CEA (ng/mL)	N.D.	6.4	9.8	4.8
	CA 19-9 (U/mL)	N.D.	7.0	11.0	6.0
3	CEA (ng/mL)	42.7	3.5	6.9	1.4
	CA 19-9 (U/mL)	8.0	30.0	67.0	6.0
4	CEA (ng/mL)	1.3	50.0	3.2	2.8
	CA 19-9 (U/mL)	25.8	2.2	40.0	33.0

Recurrent tumor was surgically resected without neoadjuvant treatment in Case 1.

CA 19-9, carbohydrate antigen 19-9; CEA, carcinoembryonic antigen; N.D., no data

**Table 5** shows the pathological findings and surgical outcomes for recurrent tumors. R0 surgery was achieved in all patients. Although only 1 patient died 48 months after surgery for recurrent tumor, the remaining 3 patients survived postoperatively.

**Table 5:** Pathological findings and surgical outcomes for recurrent tumor

Case	Pathological diagnosis	Presence of vascular invasion or minimum distance between vessel and tumor	Margin status	Complications (≥CD grade 2)	Postoperative metastasis or re-recurrence	Prognosis (follow-up period)
1	Peritoneal dissemination	No (0 mm; the tumor contacted the adventitia)	R0	None	Lung metastasis Femoral recurrence (peripheral to artificial vascular bypass)	Dead (48 months)
2	Peritoneal dissemination	No (0.7 mm)	R0	Anemia	None	Alive (69 months)
3	Local recurrence	Yes (invasion to the vessel media)	R0	Intra-abdominal abscess	Recurrence in the bladder Para-aortic lymph node metastasis	Alive (55 months)
4	No viable tumor	No (4.3 mm)	R0	None	None	Alive (21 months)

CD, Clavien–Dindo classification

## DISCUSSION

All patients underwent radical resection for the primary tumor without a positive margin, and had colorectal cancer deeper than the subserosa layer with lymphovascular invasion. Previous studies showed that risk factors for locoregional recurrence in patients with colon cancer were a more advanced disease stage or tumor grade, lymphovascular invasion, positive resection margins, left-sided tumors, bowel obstruction, perforation caused by a tumor, and invasion into adjacent structures.^4,5,12)^ All cases had at least one of these features in the present study. Three of the 4 patients had received adjuvant chemotherapy after primary tumor resection. It currently remains unclear whether adjuvant chemotherapy reduces the incidence of locoregional recurrence in Stage II/III colon cancer.^4,5)^

In our hospital, the preoperative presence of vascular invasion by the tumor was evaluated through discussions among colorectal surgeons, vascular surgeons, and radiologists based on the contact angle and length as well as the disappearance of the fat plane between the vessel and tumor on axial and coronal CT images. In histological evaluations of resected specimens, only 1 case (25%) showed true invasion of the recurrent tumor into blood vessels; the other tumors were very close to or had indistinct borders with vessels. The histological evaluations of 3 patients showed that the greatest distance between the recurrent tumor and blood vessels was 4.3 mm. Therefore, difficulties are associated with precisely assessing vascular invasion preoperatively or dissecting the narrow space between intact vessels and a recurrent tumor with clear surgical margins. In a previous case study by Abdelsatter et al. on local recurrent colorectal cancer with aortoiliac axis involvement, en bloc resection was recommended unless there was a clear dissection plane between the tumor and vascular structures during surgery.^11)^ An intraoperative margin assessment using frozen sections may be useful for confirming R0 resection.

Similar to surgery for primary colorectal cancer, R0 surgery through multivisceral resections is considered to provide a good long-term prognosis for recurrent colorectal cancer,^9,10)^ Kruschewski et al. examined 31 patients with recurrent colorectal cancer according to R0 resection; patients who underwent R0 resection had a better overall survival than those without R0 resection (R0: 106.1 months vs. R1: 72.5 months vs. R2: 37.9 months vs. inoperative: 42.2 months, *p* = 0.001).^13)^ Additionally, neoadjuvant chemotherapy in combination with multivisceral resection also resulted in higher R0 rates than surgery alone and achieved long-term survival with an overall survival rate of 75%–85% at 3–5 years.^14,15)^ Preoperative radiation alone and in combination with chemotherapy reduced recurrence rates and prolonged survival after surgery for recurrent colorectal cancer.^6,14,16)^ Preoperative CRT may be superior to postoperative treatment in terms of down-staging, clearer margins, and better local control in recurrent colorectal cancer.^17)^ In our patients, the regimen and duration of neoadjuvant treatment were determined by multi-disciplinary team meetings. Three patients received neoadjuvant chemotherapy or CRT for recurrent colorectal cancer, and all obtained negative resected margins.

There are no clear recommendations for adjuvant chemotherapy after curative resection for recurrent colorectal cancer. However, several studies reported that adjuvant chemotherapy after curative resection for metastasis, including locoregional recurrence from colorectal cancer, was associated with better prognosis.^18–20)^ In our hospital, adjuvant chemotherapy is prescribed after discussions among doctors, considering adverse events from previous chemotherapy, the patient’s general conditions, and preferences. After shared decision-making, 2 patients received adjuvant chemotherapy after curative surgery for recurrent tumors. Regarding long-term prognosis, 2 patients had metastasis or other local recurrence. However, 3 patients are currently alive without cancer.

In the aforementioned case study by Abdelsattar et al., 12 patients with recurrent tumors from colorectal cancer involving the aortoiliac axis underwent surgical resection, 7 of whom required arterial reconstruction using prosthetic vascular grafts, femoral–femoral bypass, or primary anastomosis, while 1 required venous reconstruction. Thirty-day morbidity was observed in 9 patients (75%); however, graft complications, postoperative bleeding, and 30-day mortality were not documented in their series.^11)^ We reconstruct the resected veins aiming at good lymphatic drainage and preventing deep vein thrombosis (DVT) in the early postoperative period. Moreover, postoperative edema may be caused by disruption of lymphatic drainage when the iliac or femoral veins are not reconstructed.^21,22)^ On the other hand, even when the resected veins are reconstructed, edema may develop due to graft occlusion and clotting. None of our patients developed lower limb edema or DVT in the perioperative period. When prosthetic grafts are used for reconstruction, particularly with simultaneous gastrointestinal resection, graft infection is another major concern. With deliberate case selection for prosthetic graft reconstruction, meticulous surgical techniques, careful bowel preparation, and thoughtful antibiotic prescription, the risk of graft infection may not be elevated.^23,24)^ We basically perform both mechanical and chemical bowel preparations to reduce postoperative complications in colorectal surgery, although their effects remain controversial.^25,26)^ According to a previous review, preoperative antibiotic prophylaxis is one of important strategies to prevent graft infection and should be administered within an hour of skin incision.^27)^ In addition, we perform vascular reconstruction prior to gastrointestinal resection in 1-stage operations to separate surgical fields, and change surgical instruments between vascular and gastrointestinal surgical procedures. In our cases, there were no complications associated with vascular resection or reconstruction; however, 2 patients (50%) developed other complications. Therefore, a preoperative assessment of the need for combined vascular resection and sufficient discussions on surgical strategy selected among colorectal and vascular surgeons may allow for safe en bloc resection with adjacent blood vessels and their repair. Although a staged-surgery strategy may be useful for avoiding prosthetic graft infections, the vascular bypass-first approach has disadvantages such as possible progression of recurrent tumors or new metastasis to other organs during the interval between surgeries. Moreover, graft thrombosis may develop in the bypass.

## CONCLUSIONS

We herein reported 4 patients who underwent radical resection associated with the resection of adjacent iliac or femoral vessels for colorectal cancer recurrence with favorable outcomes. Difficulties are associated with preoperatively evaluating invasion to the adjacent blood vessels. Based on a histopathological examination, it appeared to be challenging to obtain a negative resected margin in all patients without combined resection of the recurrent tumor and adjacent vessels. Therefore, surgical procedures need to be considered to perform R0 resection for the local recurrence of colorectal cancer if it is suspected to invade blood vessels.

## SUPPLEMENTARY MATERIAL

Supplementary Fig. 1 Elastica van Gieson staining of the resected recurrent tumor together with the left internal iliac artery in Case 3. Original magnification: ×20. Arrowheads indicate recurrent tumor margins near the vessel. Two-way arrows indicate the layers of the intima and media, and 1-way arrow indicates the adventitia. Blue spots were artificially marked with ink.

## ACKNOWLEDGMENTS

We would like to thank the Medical English Service (Kyoto, Japan) for their assistance with editing this manuscript.

## DECLARATIONS

### Funding

No funding was received for conducting this study.

### Authors’ contributions

Hiroaki Nozawa contributed to the study conception and design.

Data collection was performed by Kentaro Abe and Hiroaki Nozawa.

The first draft of the manuscript was written by Kentaro Abe, and the remaining authors made critical comments on it.

All authors read and approved the final manuscript and agree to be accountable for all aspects of the study.

### Availability of data and materials

The datasets supporting the conclusions of this article are included within the article and its supplementary material.

### Ethics approval and consent to participate

This study was conducted retrospectively using data obtained for clinical purposes. The present study was approved by the Ethics Committees of the University of Tokyo (No. 3252-[17]).

### Consent for publication

Informed consent was replaced by the obligation of information to the participants and their right to opt out.

### Competing interests

The authors declare that they have no competing interests.
