# Supplementary figures and images for: Recurrent Tumor in Colorectal Cancer Requiring Combined Resection of Iliac or Femoral Vessels: Report of Four Cases
Source: Surg Case Rep. 2025 May 1;11(1):24-0159. doi: 10.70352/scrj.cr.24-0159 (PMC12056520; doi:10.70352/scrj.cr.24-0159)

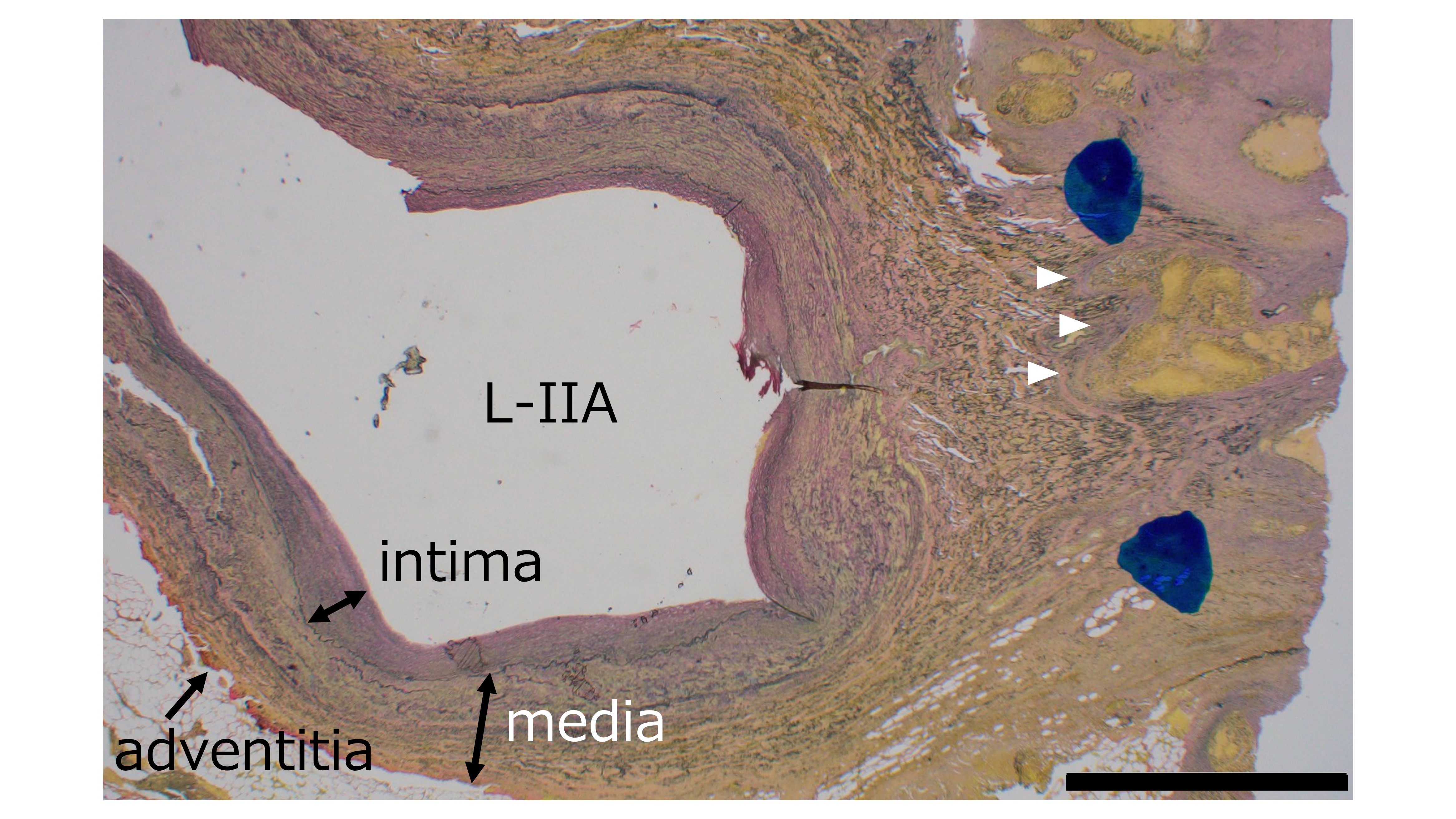

Supplement: Supplementary Fig. 1 [file scr-11-01-24-0159-s001.jpg]
